# *Hormophysa triquerta* polyphenol, an elixir that deters CXCR4- and COX2-dependent dissemination destiny of treatment-resistant pancreatic cancer cells

**DOI:** 10.18632/oncotarget.13900

**Published:** 2016-12-10

**Authors:** Sheeja Aravindan, Satishkumar Ramraj, Kathiresan Kandasamy, Somasundaram S. Thirugnanasambandan, Dinesh Babu Somasundaram, Terence S. Herman, Natarajan Aravindan

**Affiliations:** ^1^ Department of Marine Sciences, Center of Advanced Study in Marine Biology, Annamalai University, Parangipettai, TN, India; ^2^ Stephenson Cancer Center, Oklahoma City, OK, USA; ^3^ Department of Radiation Oncology, University of Oklahoma Health Sciences Center, Oklahoma City, OK, USA

**Keywords:** pancreatic cancer, seaweed polyphenols, tumor invasion and metastasis, residual pancreatic cancer, tumor relapse and recurrence

## Abstract

Therapy-resistant pancreatic cancer (PC) cells play a crucial role in tumor relapse, recurrence, and metastasis. Recently, we showed the anti-PC potential of an array of seaweed polyphenols and identified efficient drug deliverables. Herein, we investigated the benefit of one such deliverable, *Hormophysa triquerta* polyphenol (HT-EA), in regulating the dissemination physiognomy of therapy-resistant PC cells *in vitro*,and residual PC *in vivo*. Human PC cells exposed to ionizing radiation (IR), with/without HT-EA pre-treatment were examined for the alterations in the tumor invasion/metastasis (TIM) transcriptome (93 genes, QPCR-profiling). Utilizing a mouse model of residual PC, we investigated the benefit of HT-EA in the translation regulation of crucial TIM targets (TMA-IHC). Radiation activated 30, 50, 15, and 38 TIM molecules in surviving Panc-1, Panc-3.27, BxPC3, and MiaPaCa-2 cells. Of these, 15, 44, 12, and 26 molecules were suppressed with HT-EA pre-treatment. CXCR4 and COX2 exhibited cell-line-independent increases after IR, and was completely suppressed with HT-EA, across all PC cells. HT-EA treatment resulted in translational repression of IR-induced CXCR4, COX2, β-catenin, MMP9, Ki-67, BAPX, PhPT-1, MEGF10, and GRB10 in residual PC. Muting CXCR4 or COX2 regulated the migration/invasion potential of IR-surviving cells, while forced expression of CXCR4 or COX2 significantly increased migration/invasion capabilities of PC cells. Further, treatment with HT-EA significantly inhibited IR-induced and CXCR4/COX2 forced expression-induced PC cell migration/invasion. This study (i) documents the TIM blueprint in therapy-resistant PC cells, (ii) defines the role of CXCR4 and COX2 in induced metastatic potential, and (iii) recognizes the potential of HT-EA in deterring the CXCR4/COX2-dependent dissemination destiny of therapy-resistant residual PC cells.

## INTRODUCTION

With an expected 53,070 new cases in 2016, pancreatic cancer (PC) is one of the major causes of cancer death in the United States [1]. For four decades, the 5-year survival rate for PC has hovered around 5% [2]. In addition to its silent nature and tendency for late discovery, PC shows unusual resistance to chemotherapy and radiation. The use of radiation therapy (RT) in PC treatment is multi-fold: (i) RT after surgery prevents tumor relapse or recurrence, (ii) RT before surgery, along with chemotherapy, shrinks and makes removing borderline resectable tumors easier, (iii) RT can be the main treatment, combined with chemotherapy, for locally advanced and unresectable cancers; (iv) RT relieves pain in patients with advanced cancers, and; (iv) RT can be used as the prime modality for people who are not healthy enough for other treatments [3, 4]. However, clinical trials that investigated the benefit of RT, either alone or as part of chemo-RT, have shown equivocal clinical outcomes in patients with PC [4, 5]. Only 20% of primary PCs show a determinate response to RT [4, 6]. The lack of clinical efficacy of RT mandates the investigation of methods to overcome radiation resistance in PC. To that end, radio-sensitizing PC cells could significantly benefit patients diagnosed with resectable disease who suffer from local and/or distant failure post-resection [7, 8]. The need to defeat PC's induced radio-resistance and to potentiate therapeutic radiation highlights the necessity of elucidating the underlying mechanisms of PC.

Adding to the complexity, metastatic disease develops after surgery combined with pre/post radiotherapy (RT). However, the onset and orchestration of the sequential functional events of metastasis, including malignant cell release from the primary tumor site, inhabitation, and clonal expansion at distant sites, is poorly understood [9]. Initial event of metastasis originates in the primary tumor microenvironment, where intercellular molecular signaling cross talk between the tumor, surrounding stroma, and adjacent tissues leads to extracellular matrix rearrangement, cell motility enhancement, and proliferation promotion. The genetic rearrangements that occur in cancer cells drive uncontrolled growth, aberrant replication and sustained angiogenesis, and regulate anti-growth signals and evade apoptosis; these rearrangements collectively orchestrate progressive metastatic disease [10]. Based on the concept of multistage carcinogenesis in PC, continuous acquisition of these genetic rearrangements in therapy-resistant cancer cells is instrumental for post-surgical recurrence and/or metastasis. Since numerous genes are engaged in the sequential processes of tumor dissemination [11–18], understanding the comprehensive TIM transcriptome in therapy-resistant surviving PC cells is crucial. Accordingly, the objective of this study is to understand the comprehensive TIM transcriptome in therapy-resistant surviving PC cells and identify the genetic determinants that govern radio-resistance and subsequent tumor recurrence and dissemination. This study identified two such genetic determinants, CXCR4 and COX2, in a panel of radio-resistant, genetically diverse PC cells. CXCR4 is the only receptor that is expressed in the normal pancreas, PC cells, and all pancreatic tumor specimens [19]. Likewise, researchers have reported > 70% COX-2 expression in clinical PC [20–22]. We investigated the influence of radiation-induced CXCR4 and COX2 in dictating the metastatic state of surviving PC cells.

The development of drug deliverables targeting such genetic determinants is imminent and could shift the current therapeutic approach in progressive PC. The health care industry heavily relies on natural products [23], as these derivatives constitute > 50% of drugs in clinical use. In parallel to the NCI's ongoing probable drug screening of >100,000 extracts against tumor systems [24], extensive research to identify anticancer deliverables from various marine floral compounds, including seaweeds, is underway [25]. The anticancer potential of seaweeds that contain large amounts of polyphenols (catechin, epicatechin, EGCG, gallic acid) has been well documented [26, 27]. Further, studies have demonstrated the anti-proliferative [28], tumor-regressing [29], and metastasis-inhibiting [30] efficacy of seaweed polyphenols. A linear and directly proportional relationship between the anti-carcinogenic activity and antioxidant activity of polyphenols has been documented in many *in vivo* models [29, 31–33]. In assorted tumor models, researchers have recognized the benefit of seaweed extracts in effectively suppressing the functional cellular events that drive tumor progression, including tumor cell growth, cell-cycle release, proliferation, survival, DNA repair, angiogenesis, and metastasis [34–39]. Recently, our *in vitro* studies screened a panel of polyphenols derived as polarity gradient extractions from five different seaweed species for their efficacy against PC [40], and recognized three deliverables with high impact on impeding autophagy signaling [41] and PC cancer stem cell status [42]. Hence, these fractions could be clinically translatable in this setting. We hypothesize that one such fraction, *Hormophysa triquerta* polyphenol (HT-EA), will result in the inhibition of crucial genetic determinants of the TIM phenotype. Delineating such efficacy in radio-resistant PC cells will identify a ‘drug deliverable’ that not only radio-sensitizes PC cells, but will also potentiate the benefit of RT in the treatment of this deadly disease.

## RESULTS

### Radiotherapy prompts tumor invasion and metastasis transcriptome activation in resistant PC cells

To define the radio-responsive TIM-related signaling in PC cells, we investigated the alterations in mRNA levels for 93 well-characterized TIM molecules (Table S1) in genetically diverse human PC cells exposed to clinical RT. QPCR profiling revealed unique amplification signatures across treatment groups and cell lines. Profile-to-profile expression distinctions were normalized with in-house controls (HPRT-1, GAPDH, and/or β-actin), hierarchically clustered (complete linkage) with Gene Cluster (http://bonsai.hgc.jp/~mdehoon/software/cluster/software.htm), and examined using Maple Tree (v0.2.3.2 Beta, rana.lbl.gov/EisenSoftware.htm), which provides self-organizing maps of distinctive gene expression profiles for every condition and cell line investigated (Figure S1). Overall, RT resulted in the activation of 36, 53, 29, and 42 TIM molecules in surviving Panc-1, Panc-3.27, BX-PC3, and MiaPaCa-2 cells, respectively. Interestingly, cells→genes traverse analysis identified cell-line-independent activation of 10 genes (across 4 cell lines), 15 genes (in 3 cell lines), and 24 genes (in 2 cell lines).

**Figure 1:**
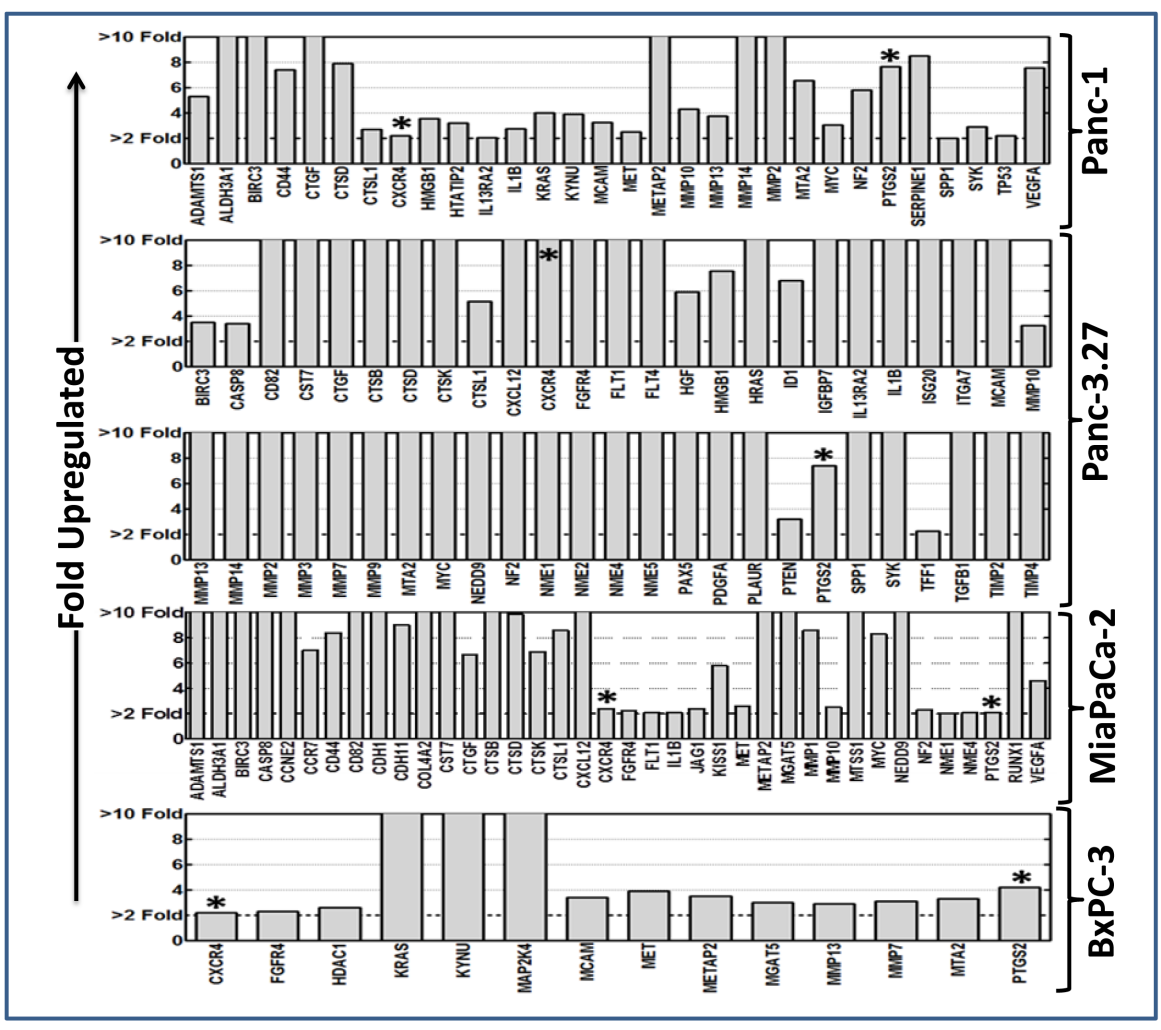
Alteration of tumor invasion metastasis transcriptome in PC cells surviving after fractionated RT Clinical doses of radiation (2 Gy/Day for 5 days for a total dose of 10 Gy) significantly induced (≥ 2 fold upregulation) tumor invasion and metastasis transcriptome in surviving *Panc-1*, *Panc-3.27*, *MiaPaCa-2*, and *BxPC-3* cells. Two genes, CXCR4 and PTGS2, showed consistent *cell-line-independent* upregulation. Quantitative transcriptional expression of 93 TIM molecules were assayed using custom-archived QPCR profiling.

Applying stringent criteria, RT significantly increased the expression of 30, 50, 15, and 38 TIM genes in Panc-1, Panc-3.27, BX-PC3, MiaPaCa-2 cells (Figure 1). Two genes, *CXCR4* and *PTGS2,* showed *cell-line-independent* upregulation after FIR exposure. Thirteen genes, *Birc3*, *CTSD*, *CTSL1*, *FGFR4*, *IL1β*, *MCAM*, *Met*, *Metap2*, *MMP10*, *MMP13*, *MTA2*, *Myc*, and *NF2* showed cell-line-independent activation in at least three cell lines. After RT, a set of 26 TIM genes (*Adamts1*, *Aldh3A1*, *kRas*, *Casp8*, *Kynu*, *CD44*, *CD82*, *CST7*, *CTSB*, *CTGF*, *CTSK*, *TP53*, *HMGB1*, *CXCL12*, *IL13Ra2*, *FLT1*, *MMP-2*, *-7*, *-14*, *MGAT5*, *NEDD9*, *NME-1*, *-4*, *VEGFA*, *SPP1*, *SYK*) were upregulated in two cell lines. Distinctively, a small subset of genes showed *cell-line-dependent* activation in Panc-1 (*HTATIP2*, *Serpine 1*), Panc-3.27 (*FLT4*, *HGF*, *Hras*, *ID1*, *IGFBP7*, *ISG20*, *ITGA7*, *MMP-3*, *-9*, *NME-2*, *-5*, *Pax5*, *PDGFA*, *PLAUR*, *PTEN*, *TDD1*, *TGFβ1*, *Tmip-2*, *-4*), BxPC-3 (*HDAC1*, *MAP2K4*), and MiaPaCa-2 (*CCNE2*, *CCR7*, *CDH-1*, *-11*, *Col4A2*, *Jag1*, *Kiss1*, *MMP1*, *MTSS1*, *Runx1*) cells. These results highlight the induction of TIM-related transcripts in radio-resistant PC cells. Further, these data identify the *cell-line-independent* and *cell-line-dependent* upsurge in TIM transcriptional responses in PC cells after RT.

### HT-EA target therapy-orchestrated onset of TIM transcription in human PC cells

**Figure 2:**
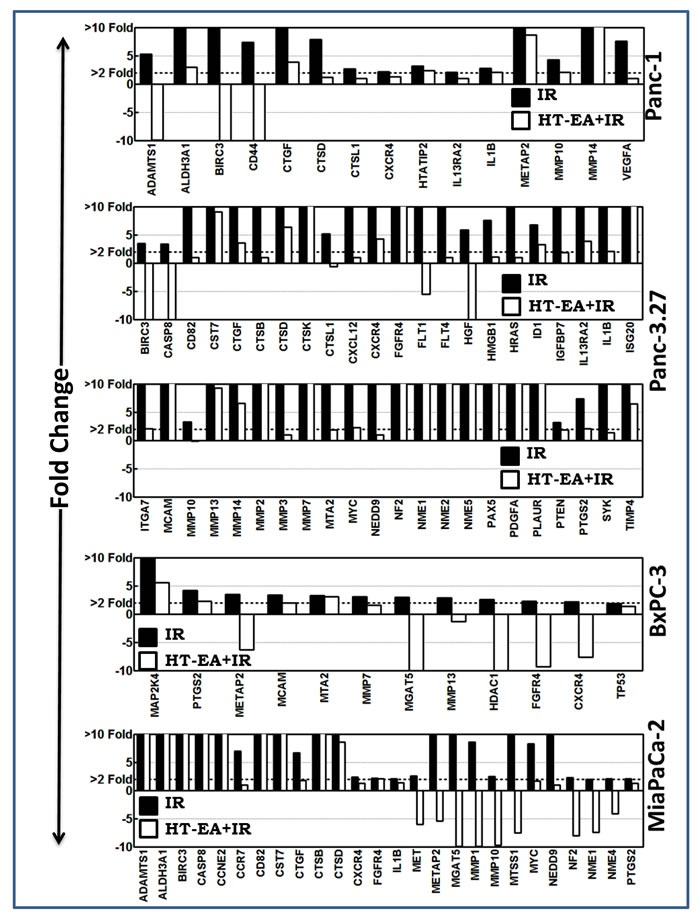
HT-EA alleviates RT-associated activation of tumor invasion and metastasis transcriptome in surviving PC cells Histograms of QPCR profiling comparison analysis showing the *HT-EA*-associated regulation of fractionated radiation-induced tumor invasion and metastasis transcriptome in surviving *Panc-1*, *Panc-3.27*, *BxPC-3*, and *MiaPaCa-2* cells.

We investigated the alterations in the transcription of TIM molecules in human PC (Panc-1, Panc-3.27, BxPC-3, MiaPaCa-2) cells that were pretreated with HT-EA and exposed to radiation. Pre-treating cells with HT-EA inhibited 15 (of 30), 44 (of 50), 12 (of 15), and 26 (of 38) FIR-induced TIM molecules in Panc-1, Panc-3.27, BxPC-3, and MiaPaCa-2 cells, respectively (Figure 2). Interestingly, treatment with HT-EA repressed radiation-induced *CXCR4* across all cell lines investigated. In addition, *PTGS2 (COX2)*, upregulated across PC cells after RT, was completely suppressed with HT-EA (Figure 2). Moreover, cell-line-independent inhibition of *Birc3*, *CTGF*, *CTSD*, *FGFR4*, *IL1β*, *Metap2*, *MMP10* (3 cell lines), *ADAMTS1*, *Aldh3A1*, *Casp8*, *CD82*, *CST7*, *CTSB*, *CTSL1*, *IL13Ra2*, *MMP-7*, *-13*, *-14*, *MGAT5*, *MTA2*, *MCAM*, *MYC*, *NEDD9*, *NF2*, and *NME-1* (2 cell lines) were observed with HT-EA treatment. Conversely, *HTAIP2*, *VEGFA* (Panc-1), *FLT-1*, *-4*, *HGF*, *HMGB1*, *HRAS*, *ID1*, *IGFBP7*, *ISG20*, *ITGA7*, *MMP-2*, *-3*, *NME-2*, *-5*, *PAX5*, *PDGFA*, *PLAUR*, *PTEN*, *SYC*, *TIMP-4* (Panc-3.27), *HDAC1*, *MAP2K4*, *TP53* (BxPC-3), *CCNE2*, *CCR7*, *MET*, *MTSS1*, and *NME4* (MiaPaCa-2) showed *cell-line-dependent* inhibition after HT-EA pretreatment.

### HT-EA regulates translation of CXCR4, COX2, and other crucial TIM targets (β-catenin, MMP9, Ki-67, NKX3.2, PhPT1, MEGF10, GRB10) in residual PC

**Figure 3:**
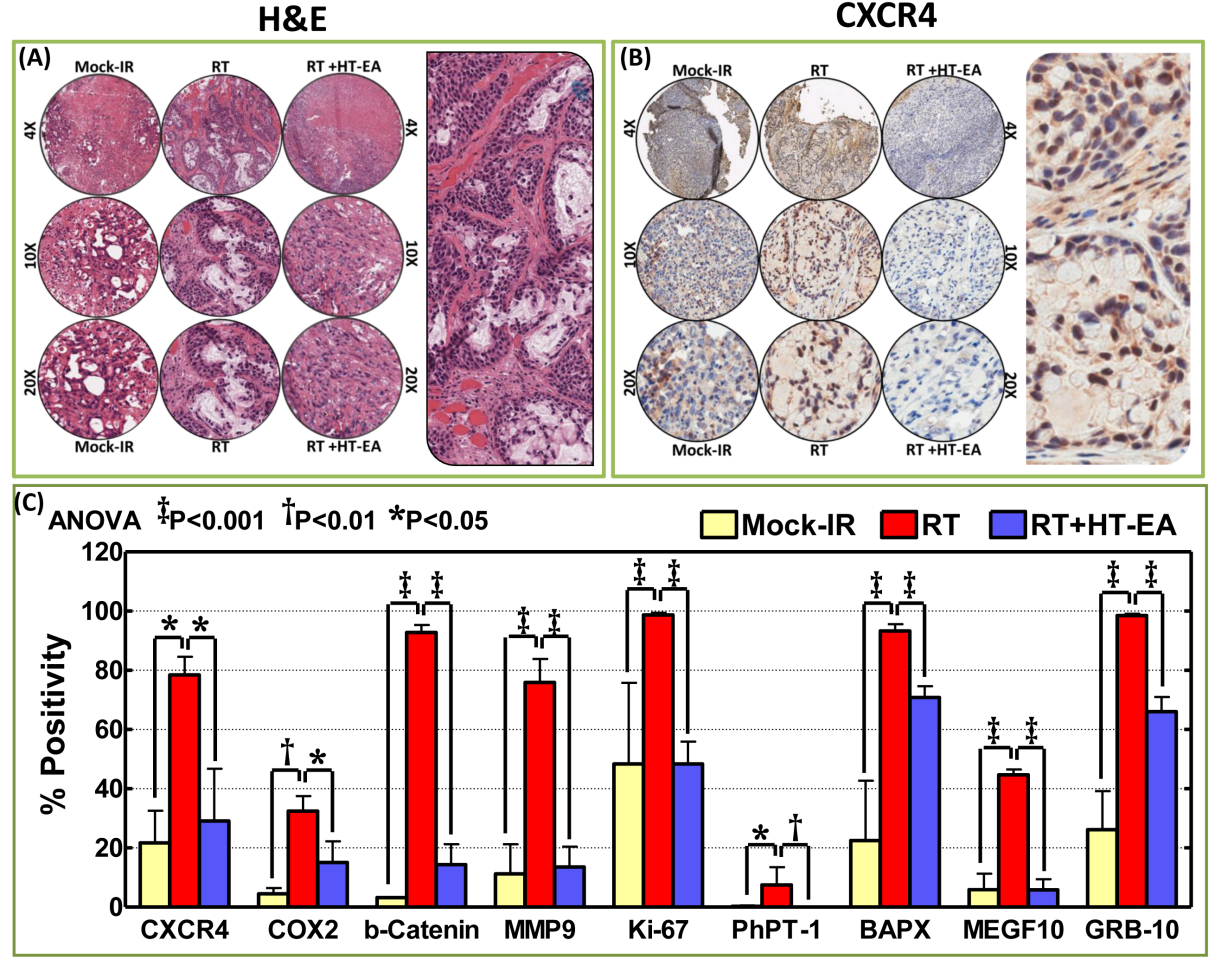
HT-EA mitigates RT-associated translation of tumor invasion and metastasis targets in residual PC **A**. Representative microphotographs from hematoxylin & eosin stained PC tissue microarray (TMA) constructed with xenografts (established from MiaPaCa-2) exposed to mock-irradiation or fractionated irradiation, with or without HT-EA treatment. Pullout shows the staining pattern (20x magnification). **B**. Representative microphotographs from CXCR4-stained PC tissue microarray (TMA) constructed with xenografts exposed to mock-irradiation or fractionated irradiation, with or without HT-EA treatment. Pullout shows the staining pattern (20x magnification). **C**. Aperio TMA quantitation analysis showing protein-specific positivity magnitudes (mean + *SD*) in mock-irradiated PC xenografts, and residual PC after clinical RT, with or without HT-EA treatment. Group-wise comparisons were made using Two-way ANOVA with Bonferroni's post-hoc correction.

**Figure 4:**
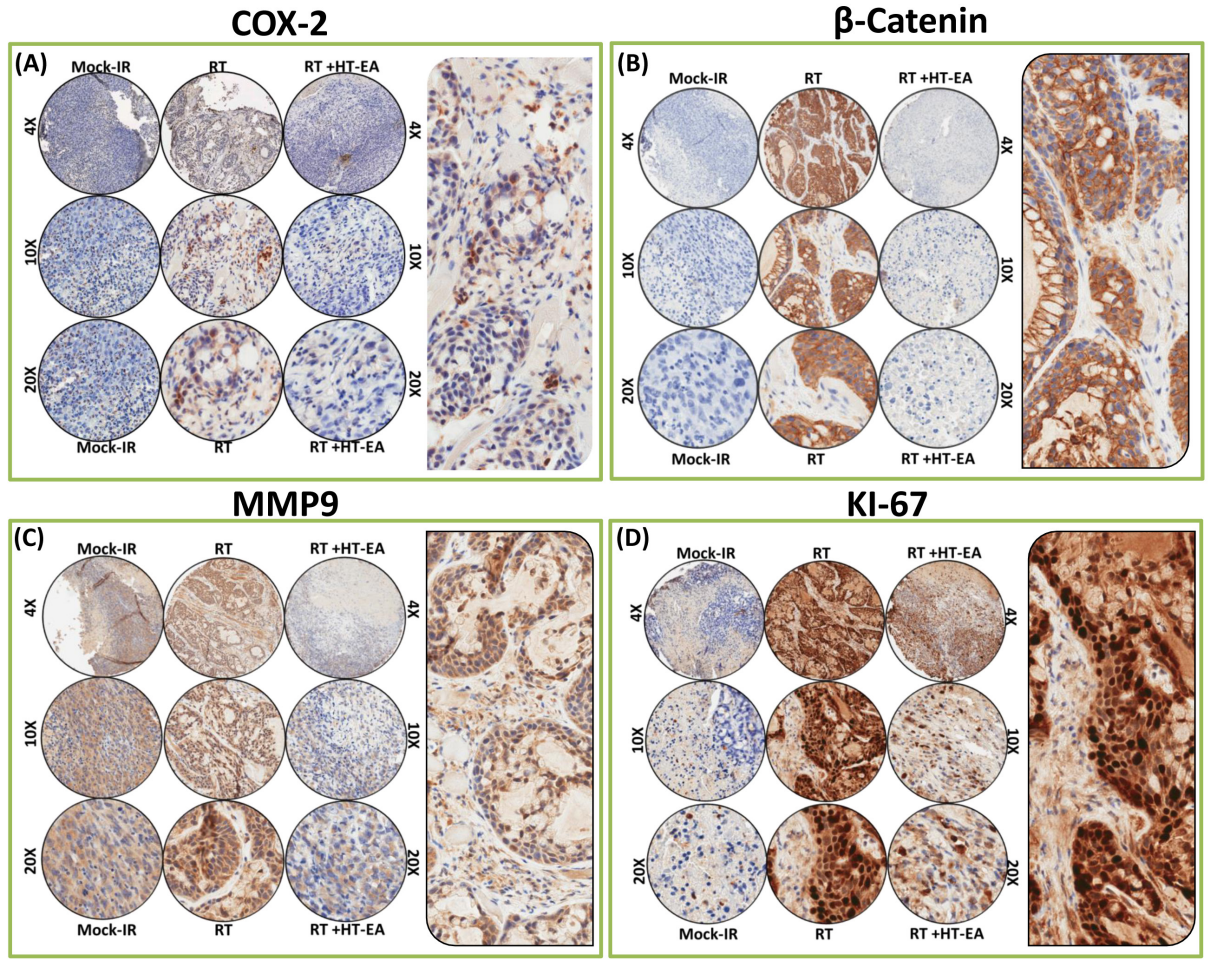
HT-EA mitigates RT-associated translation of COX2, β-catenin, MMP9, and Ki-67 in residual PC Representative microphotographs from **A**. Cox2-, **B.** β-catenin-, **C**. MMP9-, and **D**. Ki-67- stained PC tissue microarray (TMA) constructed with xenografts (established from MiaPaCa-2) exposed to mock-irradiation or fractionated irradiation, with or without HT-EA treatment. The pullout in each panel represents the corresponding Ab staining pattern in 20x magnification showing the protein-specific cellular localization. Please see Figure 3C for Aperio TMA quantitation analysis of COX2-, β-catenin-, MMP9-, and Ki-67-specific positivity magnitudes (mean + *SD*) in mock-irradiated PC xenografts, and residual PC after clinical RT, with or without HT-EA treatment.

To investigate whether HT-EA regulates radiation-induced common targets (CXCR4, COX2) and other critical proteins (β-catenin, MMP9, Ki-67, NKX3.2, PhPT1, GRB10) that are instrumental in PC progression after therapy, we examined their alterations in PC cells that were selectively exposed to RT, with or without a daily dose of HT-EA. IHC staining consistency across samples was achieved by TMA construction (Figure 3A) utilizing histopathological evaluations of individual H&E stained tumor tissues, coupled with automated IHC. C-X-C chemokine receptor type 4 (CXCR4) IHC staining exhibited baseline positivity in mock-irradiated controls (Figure 3B & 3C). Selective CXCR4 localization was observed in the plasma membrane (see pullout in Figure 3B). We observed no staining when the no-primary negative control for CXCR4 was used. IHC revealed strong positivity and abundant presence (~80% of cells) of CXCR4 in residual PC after RT (Figure 3B & 3C). However, HT-EA treatment completely (*P* < 0.05) suppressed CXCR4 in residual PC (Figure 3C). Figure 4A portrays the expression profiles of COX2 (PTGS2) in PC exposed to clinical FIR (compared with mock-IR controls), with or without HT-EA treatment. Compared with the stromal cells, we observed strong COX2 positivity in tumor cells. At the sub-cellular level, COX2 is abundant in cytoplasm, with weak membranous localization (see pullout in Figure 4A). Although COX2 was detectable under all conditions, strong (*P* < 0.01) and abundant positivity was observed in residual PC (Figure 3C). There was a substantial variance in COX2 expression between the group that received radiation alone and the group that received radiation and HT-EA (Figures 3C & 4A). These modifications in CXCR4 and COX2 expression in response to RT in the presence and absence of HT-EA treatment corroborated well with the transcriptional modifications observed with QPCR profiling.

β-catenin (Cadherin-associated protein and beta 1) IHC staining revealed predominant plasma membrane localization (see pullout in Figure 4B). Compared with the baseline expression in mock-IR controls, we observed strong and abundant ( > 90% of the cells) β-catenin expression (*P* < 0.001) in residual PC after clinical RT (Figures 3C & 4B). β-catenin immunoreactivity was completely reduced *(P* < 0.001) to near basal levels (Figure 3C & 4B) in all PC cores from HT-EA-treated animals. Figure 4C shows representative IHC results for Matrix metallopeptidase 9 (MMP-9) in PC cells that received RT, with or without HT-EA pre-treatment. MMP-9 expression was stronger and selective in the cytoplasm, with some weak positivity in the nucleus (see pullout in Figure 4C). While mock-IR controls exhibited measurable levels of MMP-9, we observed strong MMP-9 positivity (*P* < 0.001) in residual PC after RT. Weak positive MMP-9 expression was observed with HT-EA treatment (Figure 3C & 4C). Likewise, Ki-67 positivity was detected under all conditions. IHC staining revealed a strong nuclear localization (see Figure 4D pullout). Ki-67 positivity ranged from 48.35% in mock-IR control to 98.70% of residual tumors (Figure 3C). HT-EA treatment produced fewer Ki-67-positive epithelial cells (48.38%; Figures 3C and 4D).

**Figure 5:**
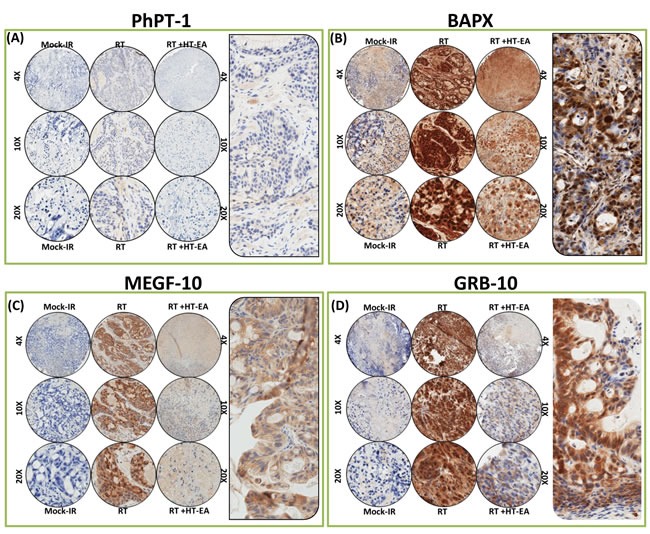
HT-EA mitigates RT-associated translation of PhPT-1, BAPX, MEGF-10, and GRB-10, in residual PC Representative microphotographs from **A**. PhPT-1, **B**. BAPX, **C**. MEGF-10, and **D**. GRB-10-stained PC tissue microarray (TMA) constructed with xenografts (established from MiaPaCa-2) exposed to mock-irradiation or fractionated irradiation, with or without HT-EA treatment. The pullout in each panel represents the corresponding Ab staining pattern in 20x magnification, showing the protein-specific cellular localization. Please see Figure 3C for Aperio TMA quantitation analysis of PhPT-1-, BAPX, MEGF-10-, and GRB-10-specific positivity magnitudes (mean + *SD*) in mock-irradiated PC xenografts, and residual PC after clinical RT with or without HT-EA treatment.

The PhPT1 (Phospho-histidine phosphatase 1) IHC staining profile for PC specimens after RT, with or without HT-EA treatment, are presented in Figure 5A. No traceable PhPT1 immunoreactivity was observed in control (mock-IR) conditions, consistent with the documented low expression of PhPT1 in PC ( www.proteinatlas.org). Radiation-exposed PC cells revealed a detectable, yet weak, cytoplasmic localization of PhPT1 (see Figure 5A pullout). However, RT-associated induction in PhPT1 localization was completely reduced with HT-EA treatment (Figures 3C & 5A). IHC analysis of BAPX1 (NK3 homeobox 2, NKX3.2) revealed baseline positivity in mock-IR controls. Bapx1 was mostly localized in the nucleus (see Figure 5B pullout). After RT, Bapx1 expression was strongly positive and present in 93.92% of the PC cells lingering after treatment (Figure 3C & 5B). Conversely, HT-EA treatment in conjunction with RT resulted in a significant (*P* < 0.001) reduction in Bapx1 levels in residual PC (Figure 3C & 5B).

Likewise, cellular localization levels of Multiple EGF-like-domains 10 (MEGF10) were examined in PC xenografts exposed to RT, with and without polyphenol treatment (Figure 5C). MEGF10 immunoreactivity showed marginal positivity in control (mock-IR) specimens (Figure 3C), consistent with reported levels in human PC (www.proteinatlas.org). Conversely, the residual PC cells exhibited up to 44.71% immunoreactivity for MEGF10 localization. MEGF10 positivity appeared brown in color, and was mostly localized in the plasma membrane in a spotted pattern (see Figure 5C pullout). We observed a significant (*P* < 0.001) inhibition of induced MEGF10 expression in residual PC tissues with HT-EA treatment (Figures 3C & 5C). Further, we examined GRB10 (Growth factor receptor-bound protein 10), a protein that is highly expressed in human PC (www.proteinatlas.org), in PC exposed to RT, with and without HT-EA treatment (Figure 5D). Mock-irradiated PC tumors showed low GRB10 immunoreactivity. GRB10 positivity was intense and present in 98.51% of the PC cells that survived RT (Figure 3C). GRB10 positivity appeared granular and dark brown in color, and was primarily localized in the cytoplasm (see Figure 5D pullout). Treatment with HT-EA significantly (*P* < 0.001) reduced the radiation-induced GRB10 expression in PC tissues. These observations demonstrate the efficacy of HT-EA in mitigating therapy-responsive induction of β-catenin, MMP9, Ki-67, NKX3.2, PhPT1, GRB10, and MEGF10 in residual PC.

### HT-EA deters the radiotherapy-induced CXCR4/COX2 facilitated metastatic state of the resistant PC cells

**Figure 6:**
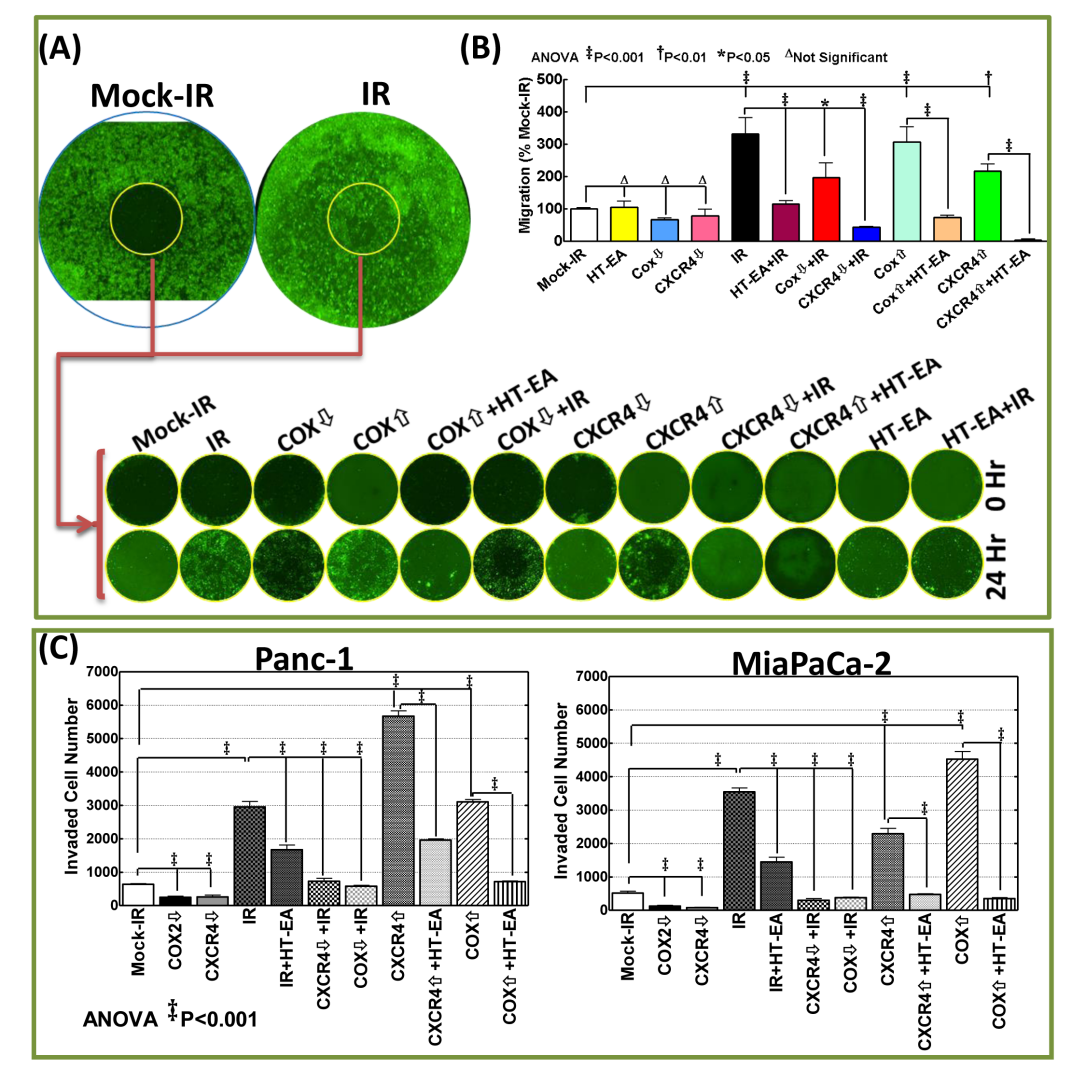
RT-associated activation of CXCR4 and COX-2 determined the metastatic state of the surviving PC cells **A**. Representative whole images of cell migration assay (mock-IR and IR) and the representative images of migrated cells in the cell seed stopped areas (wound) taken after 0 and 24h of human Panc-1 cells exposed to either mock-irradiation or radiation with and without HT-EA treatment, forced expression of COX2 or CXCR4 with/without HT-EA treatment, after silencing COX2/CXCR4, after HT-EA treatment alone or, after silencing COX2/CXCR4 and exposed to FIR. **B**. Panc-1 cells migrated to seed stopped area (wound) and **C**. Matrigel-invaded MiaPaCa-2 and Panc-1 cells (mean + *SD*) showing 1) heightened migratory and invasive potential of cells that survive RT; 2) complete inhibition of IR-induced migration/invasion potential with HT-EA treatment, by muting IR-induced CXCR4 or COX2; 3) high migration/invasion capacity in CXCR4 or COX2 expressed cells, and; 4) complete inhibition of CXCR4-/COX2-induced migratory/invasive capabilities with HT-EA treatment. Expression of CXCR4 or COX-2 (Origene) was carried out using TurboFectin 8.0 and CXCR4 or COX-2 silencing with shRNA (MISSION^®^ shRNA, Sigma-Aldrich) following standard protocols. Invasion assays were performed using the BD Matrigel invasion assay, following standard protocols. Quantification of invaded cells was performed using Image Quant colony count analysis software. Group-wise comparisons were examined by ANOVA with Bonferroni's post-hoc corrections using GraphPad PRISM software. A *P* value of < 0.05 was considered significant.

Delineating whether RT-associated transcriptional→translational increase of CXCR4 and COX2 mediates the metastatic state of the surviving PC cells, and whether treatment with HT-EA deters such a response, we examined the associated modifications in two hallmarks of metastasis coupled with gene knock-in/knock-out approaches. For this, human PC (MiaPaCa-2, Panc-1) cells exposed to either mock-IR or IR, with or without HT-EA treatment, were examined for modulations in tumor cell migration and invasion capabilities. To define the role of COX2 and/or CXCR4 in the IR-induced metastatic state of the cells, COX2- or CXCR4-silenced PC cells were then exposed to mock-IR or IR. To identify whether HT-EA affects PC cell dissemination destiny by selectively targeting CXCR4/COX2, we assessed the alterations in tumor cell migration and invasion after ectopic expression of CXCR4/COX2 in PANC-1 and MiaPaCa-2 cells treated with HT-EA. Cell migration analysis under proliferation-controlled conditions revealed marginal migration in mock-irradiated controls of Panc-1 (Figure 6A & 6B) cells. However, we noticed a profound surge in the migration of cells that survived IR exposure, as evidenced by the complete disarray of the cells. It is pertinent to mention that, since the experiments were performed under strict proliferation control conditions, the complete disarray of the wound represents the robust movement of the cells (Figure 6A & 6B). Interestingly, treating these cells with HT-EA completely reversed radiation-induced cell migration (Figure 6A & 6B) and demonstrates the tumor cell migration inhibition potential of HT-EA in this setting. Silencing CXCR4 or COX2 remarkably reversed the induced cell migration in surviving cells (Figure 6A & 6B), identifying the definitive role of IR-induced CXCR4 and/or COX2 in tumor cell migration. Conversely, forced expression of CXCR4 or COX2 simulated the RT response, with complete disarray of cells (Figure 6A & 6B), defining their direct role in induced cell migration. Treating the cells with HT-EA reverted the forced expression-induced cell migrations (Figure 6A & 6B), demonstrating that HT-EA deters tumor cell migration by selectively targeting radiation-induced CXCR4 and COX2.

Results from a matrigel invasion assay identified heightened tumor cell invasion in RT-resistant MiaPaCa-2 and Panc-1 Cells (Figure 6C). However, pretreating these cells with HT-EA significantly (*P* < 0.001) inhibited therapy-induced invasion. Further, muting COX2 or CXCR4 completely (*P* < 0.001) deterred the induced invasion capabilities of surviving PC cells, highlighting their intrinsic therapy-associated switch in the metastatic state. Moreover, forced expression of CXCR4 and COX2 resulted in a significant increase in tumor cell invasion (Figure 6). Treating the cells with HT-EA reversed the COX2 or CXCR4 expression-associated increase in tumor cell invasion. Together, these data demonstrate that HT-EA selectively targets RT-induced CXCR4/COX2-dependent heightened invasion in surviving PC cells.

## DISCUSSION

In cancer patients, metastasis remains the major cause of death [43, 44]. Patients with PC present with progressive metastatic disease at diagnosis, with minimal response to chemotherapy and RT [45]. However, the onset of PC metastasis, particularly after first-line therapy, and the molecular mechanisms that underlie therapy resistance, tumor progression, and dissemination are poorly understood. Identifying drug deliverables that can target tumor progression after therapy will have a momentous impact on the treatment of PC. The results presented here demonstrate the initiation of a robust invasion/metastasis signaling response in the PC cells that survive RT. Studies have demonstrated the existence of functional interlinks between the sequential cellular events, such as signaling response, DNA damage recognition, and induced repair, in PC cells that survive therapy. Surviving tumor cells after RT exhibit initiation of hyperactive (oncogenic) survival and activation of the invasion/metastasis signal transduction pathways [46–58].

The results present a blueprint of the modulation of tumor cell invasion/metastasis transcriptome (93 genes) in human PC cells after clinically relevant, fractionated RT. The data show the complex pattern of TIM transcriptome elevation after RT, with a clear indication of a ‘cell-line-independent’ response. Further, we demonstrated significant clinical benefits of a seaweed polyphenol fraction in mitigating the therapy-associated activation of TIM transcriptomes *in vitro* and functional cellular localization of crucial players *in vivo*. Our study examined (a) a comprehensive panel of TIM signaling molecules after clinical RT in a panel of genetically diverse PC cells, (b) the potential of a proven anti-PC bioactive fraction of seaweed polyphenols in targeting the therapy-associated TIM transcriptome, (c) the cellular localization of key functional tumor invasion and metastasis responses driving CXCR4, COX2, β-catenin, KI-67, NKX3.2, GRB10, MEGF10, PhPT-1, and MMP-9 in established PC residual tumors after RT, (d) the clinical benefits of HT-EA in reverting the therapy-associated regulation of β-catenin, KI-67, NKX3.2, GRB10, Megf10, PhPT-1, and MMP-9 in residual tumors, (e) the intrinsic influence of radiation-induced COX2 and CXCR4 in the heightened metastatic state of the PC cells that survive radiation, and (f) the efficacy of HT-EA in selectively inhibiting radiation-induced COX2- and/or CXCR4-dependent heightened tumor cell migration and invasion. The 93 genes that we examined are functionally characterized as pro-invasion and -metastasis molecules. Hence, upregulation of these genes reflects the functional response. To that end, here we focus the discussion on gene profiles and TIM signature, with an in-depth assessment of cell-line-independent candidates and the benefits of HT-EA as an anti-metastatic drug deliverable.

Metastasis, the process by which tumor spreads or disseminates to distant organs, is a well-programmed, tightly regulated, complex, sequential process. Local invasion, the first step, requires cell release that defies cellular adhesion and prompts cell motility, which facilitates tumor cell invasion into the adjacent tissues. This is followed by the invasion of cancer cells through the basal membrane, blood, and lymphatic vessel endothelium (intravasation), thus entering the circulation. In combination with circulating leukocytes and platelets, the surviving circulating tumor cells assemble tumor emboli and are arrested at remote locations. At these sites, tumor cells invade out of the vessels (extravasation) and occupy the distant site. The continuous ongoing acquisition of genetic and epigenetic changes in these tumor cells, coupled with favorable microenvironment conditions, prompts cell proliferation and the angiogenic response that could lead to the development of metastatic tumor.

Our results document a comprehensive, cell-line-independent induction of TIM molecules in human PC cells after RT. Activation profiles of TIM molecules were fairly high in Panc-3.27 cells compared with other cell lines, presumably due to the elemental variations in radiation-response between these genetically diverse PC cells. Although researchers have recognized the instrumental role of select TIM molecules in PC, here for the first time, we report the comprehensive alterations of TIM molecules in surviving PC cells. Two genes, *CXCR4* and *COX-2*, stood out after IR. Induction of these two molecules identified them as the prime mechanisms that could drive PC metastasis after therapy. A number of additional molecules that showed elevated activation after RT in > 2 cell lines are also potential targets for sensitization.

The critical role of CXCR4 in influencing every stage of metastasis, including tumor cell migration, growth, invasion, and angiogenesis, has been recognized [19, 59–62]. Further, studies have identified that CXCR4 is the only receptor that is expressed in healthy human pancreas, PC cells, and 100% of PC specimens [19]. CXCR4 positivity and/or its heightened expression have been shown to significantly correlate with PC progression and dissemination and with a substantial reduction in OS [63]. The present study confirms a consistent and *cell*-*line-independent* transcriptional activation of CXCR4 in PC cells surviving first-line RT. Although CXCR4 is mutagenic for PC and other tumor cells [60], a recent study demonstrated that CXCR4 could also amplify the metastatic state of PC cells [19]. Consistently, silencing CXCR4 has been shown to inhibit cancer migration [64], including migration in PC [63]. More importantly, blocking CXCR4 has been shown to hinder PC carcinogenesis by impeding the cell cycle and cell colony formation [63]. Herein, our results demonstrated that CXCR4 transcription is activated in all genetically diverse PC cells that survive RT, corresponding with a translational increase in residual PC tumors, and that radiation-induced CXCR4 mediates the migration and invasion of surviving PC cells. New to science, these findings also identify that HT-EA can not only cause a significant and consistent inhibition of CXCR4 transactivation and translation in therapy-resistant PC cells, but also deters the CXCR4-dependent alterations in the metastatic state of the cells. In this regard, HT-EA targeting CXCR4 could be highly beneficial in the mitigation of metastasis after therapy and/or the onset of secondary oncogenesis in this setting.

Evidence suggests an important role of COX-2 in the initiation and evolution of many tumors [21, 65–69], including PC [20, 21]. COX-2, a catalytic enzyme that plays a critical role in the synthesis of prostaglandins [70], has been shown to be upregulated in > 70% of human PC [20–22]. In many cancers, heightened COX-2 levels have been shown to correlate with high levels of intratumoral prostaglandin E2 (PGE2) [71–73]. PGE2 was recently shown to upregulate invasive potential [74]. Studies demonstrating the activation of matrix metalloproteinases and the associated increase in invasive physiognomies with ectopic expression of COX-2 further revealed the influence of COX-2 in tumor invasion [75]. Most studies have examined the role of COX-2 in PC tumorigenesis and progression. However, to our knowledge, the current study is the first observation demonstrating the therapy-triggered activation of COX-2 in surviving cells. The results presented here established a consistent increase in COX-2 activation in all PC cell lines investigated. Biologically, COX-2 upregulation has been shown to resist programmed and/or induced cell death [76], to dictate the metastatic state in cancer cells [75], and to stimulate angiogenic events [77]. Collectively, these COX-2 upregulation-driven biological events play critical roles in tumor evolution. Our results demonstrating elevated COX-2 expression in all PC cell lines after IR and in residual tumors recognize COX-2 as a definitive target for radio-sensitization. Moreover, our findings show that induced COX-2 mediates, at least in part, the tumor cell metastatic potential. Demonstrating the efficacy of HT-EA in inhibiting the COX-2-dependent heightened metastatic state of therapy-resistant PC cells defines the benefit of this fraction in effective prevention and/or treatment of PC relapse and recurrence.

Although the benefits of seaweed constituents in cancer treatment are beginning to be discovered, ours is the first group to show the benefits of sequentially extracted polyphenol fractions in cancer medicine, particularly as treatments against PC [40]. Utilizing a comprehensive approach, we identified three fractions with high levels of activity: SA-EA, PT-EA, and HT-EA [41, 42]. Our results further demonstrate the advantage of one such promising fraction in preventing PC invasion and metastasis when used in conjunction with RT, by selectively targeting the therapy-driven molecular switches. Strikingly, HT-EA attenuated the CXCR4 and COX-2 molecules in therapy-resistant cells. Upon close examination of the data, HT-EA was noted to have a unique inhibitory signature on every panel of RT-induced cell-line-independent TIM molecules (see Results). Taken together, the data indicate that seaweed extract prompts a direct and definite inhibitory effect on invasion and metastasis in residual PC settings. This work provides a foundation for more detailed mechanistic investigations and translation into the clinic setting.

We utilized a preclinical PC residual tumor model that incorporates clinically mimicking fractionated RT selectively delivered to human PC xenografts, to identify HT-EA's efficacy in the regulation of TIM functional response molecules. To our knowledge, this is the first attempt of such an approach to determine the therapeutic response in PC. We observed increased expression of β-catenin, a crucial player in canonical Wnt signaling, in residual PC tumors. Studies have demonstrated the critical role of β-catenin in cancer cell proliferation, dissemination, and other key functional events [78–80]. The activation of β-catenin and subsequent Wnt pathway signaling reinforces the metastatic state (tumor cell migration, invasion, adhesion) of the cancer cells [79, 81, 82]. Much evidence has demonstrated the association of heightened β-catenin expression with tumor evolution and poor clinical outcomes [83–86]. In PC, aberrant β-Catenin expression has been observed and has been shown to have clinical significance with lymph node metastasis. Consistent with previous estimates, Ki-67-positive nuclei clustered heavily in the residual tumors [9,10]. Studies have shown that heightened Ki-67 levels in tumors could directly correlate to reduced time to disease progression after primary therapy, and low OS after systemic (metastatic) recurrence [87]. Furthermore, researchers have reported that a high Ki-67 level in tumors may serve as a stand-alone prognostic factor for progressive disease with metastasis [88], suggesting that metastatic cancers with low and high Ki-67 expression have different median times to progression during therapy.

In residual PC settings, we also identified novel targets, including NKX3.2, PhPTP, MEGF10, and GRB10, that could drive the therapy response in surviving cells. These proteins have not yet been characterized in PC metastasis. However, for the first time, we identified their localization in residual tumors after therapy. These proteins could play a role in orchestrating invasion and metastasis. For example, GRB10, an adaptor protein, has been shown to exhibit Bad-dependent interaction with Raf-1 kinase [89]. Nevertheless, the influence of GRB10 in activating or inhibiting signal transduction and the subsequent functional consequences *in vivo* remain uncertain. Current *in vitro* data from independent studies equivocally support both causal and regulatory roles [90–96]. MMP-9 has been extensively investigated in tumor metastasis, including PC metastasis. Evidence suggests a directly proportional relationship with elevated overexpression of MMP9 in invasive tumors and their clinical impact on tumor relapse. Nonetheless, HT-EA investigated in this study resulted in the regulation of therapy-associated increased localization of β-catenin, KI-67, NKX3.2, GRB10, Megf10, PhPT-1, and MMP-9 in residual PC models. Our results highlight the molecular blueprint of therapy-induced metastatic signaling and the clinical efficacy of HT-EA in this setting.

The limitation of this study is the use of the PC xenograft residual model. The authors are aware that orthotropic or spontaneous PC models are appropriate to elucidate the molecular events pertaining to invasion and metastasis that occur in tumors and the tumor microenvironment in response to RT. However, the present study was conducted primarily to recognize whether any radio-response in surviving tumor cells activates the metastatic state and to identify any genetic determinants that govern such a phenotype. The xenograft residual PC model utilized in the present study, coupled with the achievability of tumor-specific RT, allowed us to define such function-focused molecular events in PC and recognized the critical role of two candidates, CXCR4 and COX2, in this setting. Armed with this information, our future studies will focus on elucidating the appropriate molecular events in TME with clinically translatable spontaneous PC models that are currently under development in our laboratory.

In conclusion, for the first time, the present study reveals the molecular blueprint for tumor invasion and metastasis in PC cells that survive a course of RT, in *in vitro* (utilizing a panel of genetically diverse human PC cells) and *in vivo* (mouse model of residual PC) settings. Further, the results identified two crucial functional targets, CXCR4 and COX-2, that determine the metastatic state of the surviving PC cells. New to science, the results identified that a high-polarity extraction of seaweed polyphenol, HT-EA, not only mitigates therapy-associated onset of the TIM transcriptome in surviving PC cells and residual tumors, but also selectively targets radiation-induced CXCR4-/COX-2-dependent dissemination destiny of surviving PC cells. Together, the results identified a potential clinically translatable drug deliverable, HT-EA, that could serve as a panacea for PC progression, relapse, and recurrence. Further studies with appropriate residual PC models (spontaneous PC animal models coupled with current clinical treatment modalities), drug pharmacokinetics, and systemic tumor-targeted delivery approaches are warranted and are currently in progress in our laboratory.

## MATERIALS AND METHODS

### Cell culture

Human Panc-1 (ATCC-CRL1469), MiaPaCa-2 (ATCC-CRL1420), Panc-3.27 (ATCC-CRL2549), and BxPC-3 (ATCC-CRL1687) cells were obtained from Dr. Daniel J. Brackett (Department of Surgery, University of Oklahoma Health Sciences Center, Oklahoma City, OK). Low passages of cells were cultured and maintained as described earlier [40, 97]. For passaging and for all experiments, the cells were detached using 0.25% trypsin/1% EDTA, resuspended in complete medium, counted electronically using a Countess automated cell counter (Carlsbad, CA, USA), and incubated in a 95% air/5% CO_2_ humidified incubator.

### Xenotransplantation mouse model

All experiments conformed to American Physiological Society standards for Animal Care, carried out in accordance with the guidelines laid down by the National Research Council, and were approved by our Institutional Animal Care and Use Committee. Seven-week-old male athymic NCr-*nu/nu* nude mice (NCI, Frederick, MD) weighing 25-30 g were acclimated for at least 3 days before the study. Human MiaPaCa-2 (5×10^6^) cells suspended in 30% Matrigel (BD Biosciences) were administered SC into the right flanks. Tumor growth was periodically monitored and tumor volume was calculated using the formula volume = [(π/6) × length × width^2^]. Tumors were allowed to grow to a volume of at least 100 mm^3^. Six animals were used in the pretreatment group. Animals were randomly allocated to each group [98–102]. At the end of each experiment, animals were euthanized by CO_2_ asphyxiation, and the xenografts were harvested and subjected to downstream end-point analysis.

### *In vitro* and *in vivo* irradiation procedures

In the radiation experiments, cells were exposed to either mock-irradiation or fractionated irradiation (FIR) of 2 Gy/day for 5 days of low-LET using a Gamma Cell 40 Exactor (Nordion International, Inc., Ontario, Canada) at a dose rate of 0.81 Gy/min. *In vivo* PC xenografts were selectively exposed to clinically relevant FIR, (2 Gy/day for 5 days per week for a total of 3 weeks), to a total dose (TD) of 30 Gy. A specially designed cerrobend shield was used to encase the bodies of the mice, exposing only the flank tumors, as described earlier [53, 58, 103]. Mock-irradiated animals were treated identically, except they were not subjected to radiation.

### Polarity gradient polyphenol(s) extraction and cell treatments

Cells were plated in 100-mm tissue culture plates containing 6 ml of complete growth medium and were allowed to grow up to 70-80% confluence. Polarity-based extractions of seaweed polyphenol fractions were performed as described in our earlier studies [40]. We selectively examined ethyl acetate fraction of *Hormophysa triquerta* (HT-EA). In all *in vitro* investigations, cells were treated with 100 µg/ml of polyphenol, while a corroborated daily (*i.v*.) dose of 10 mg/Kg concentration was used for *in vivo* studies.

### Quantitative TIM transcriptome profiling

Total RNA extraction and real-time QPCR profiling were performed as described earlier [54], using a custom-made TIM transcriptome profiler ( Realtimeprimers.com, Elkins Park, PA). We started with this highly selective QPCR profiler instead of an all-encompassing gene array because the selected genes entail a well-characterized profile governing TIM. This selection facilitates interpretation of data, simplifying data acquisition and analysis, and avoids genes that are not functionally characterized. The ΔΔ^ct^ values were calculated by normalizing the gene expression levels to the expression of the housekeeping genes. The normalized data were then compared between groups, and the relative expression level of each gene was expressed as fold change. When comparing each gene's signal intensity between groups, we used a ≥ 2 fold increase or decrease to represent “stringent” criteria for upregulation or downregulation, and an increase/decrease of < 2 fold to represent “less stringent” criteria. Classifying gene regulation criteria in this way can provide an index of reliability of the gene expression data [104].

### Tissue microarray construction and quantitative immunohistochemistry

All mouse tissue microarray construction procedures and automated immunohistochemical (IHC) staining were performed in the Stephenson Cancer Center - Cancer Tissue pathology core, as described earlier [41, 42]. For the present study, slides were immunostained for COX2 (Abgent), CXCR4 (Spring Bioscience) GRB10, MEGF10 and MMP9 (Santa Cruz), BAPX1, KI-67 (Thermo Scientific), β-catenin, and PHPT1 (Cell Signaling Technology). Appropriate tissue morphologic/pathologic (H&E) controls and negative controls without primary antibody were examined in parallel. The slides were micro-digitally scanned using an Aperio Scanscope (Aperio Technologies, Inc., Buffalo Grove, IL) slide scanner. Virtual slides were constructed with digital histology. This allows assembly of tissue collections in TMA with variable magnifications. The digital images were then analyzed using Aperio Integrated Spectrum software, the web-based database system. Group-wise comparisons were made using ANOVA with Tukey's post-hoc correction. A *P* value of < 0.05 was considered statistically significant.

### Plasmid preparation and DNA/sHRNA transfection

The plasmid constructs of COX-2 (untagged-Human prostaglandin-endoperoxide synthase 2, Origene) and CXCR4 (untagged-Human chemokine [C-X-C motif] receptor 4, Origene) were amplified and purified following the manufacturer's protocol. Plasmid DNA transfections were carried out using TurboFectin 8.0 reagent (Origene) as described earlier [105]. COX2 silencing of was achieved with MISSION COX-2 shRNA (CCG GCT ATC ACT TCA AAC TGA AAT TCT CGA GAA TTT CAG TTT GAA GTG ATA GTT TTT G), while CXCR4 silencing was achieved using MISSION CXCR4 shRNA (CCG GGC GTG TAG TGA ATC ACG TAA ACT CGA GTT TAC GTG ATT CAC TAC ACG CTT TTT G; Sigma-Aldrich). Transfected cells were either mock-IR, exposed to IR, or treated with HT-EA, and were utilized for determining tumor cell invasion and migration.

### Real-time wound-healing assay

ORIS™ cell migration assay (Platypus Technologies, Madison, WI) was used to examine the alterations in PC cell migration in response to the mock-irradiation, IR exposure with/without HT-EA treatment, IR exposure with/without COX-2/CXCR4 silencing, and forced expression of COX-2/CXCR4, with/without HT-EA treatment following manufacturer's protocol. Induced or associated modification in cellular proliferation was arrested using Mitomycin C (10 μg/ml, Sigma). Cells were stained with CellTracker™ CMFDA (5-chloromethylfluorescein diacetate), Thermo Fisher Scientific, Waltham. MA), a Green-fluorescent dye, and were imaged in real-time at 0h and at 24 h using an Operetta high-content confocal immunofluorescence imager (Perkin Elmer, Inc., Waltham, MA).

### Matrigel invasion assay

For invasion assays, 1× 10^5^ cells were plated in the top chamber with a Matrigel-coated membrane (24-well insert; pore size, 8 μm; BD Biosciences). Cells were plated in medium without serum, and medium supplemented with serum was used as a chemoattractant in the lower chamber. The cells were incubated for 24 h. Cells that did not migrate or invade through the pores were removed by a cotton swab. Cells on the lower surface of the membrane were fixed with 3:1 methanol: acetic acid and stained using 0.1% Crystal violet to visualize the invaded cells.

SA, SR, DS and NA performed the experiments and contributed to the acquisition of the data.

NA, SA, and SR contributed to data analysis and interpretation.

NA and SA drafted the manuscript, and KK, ST, and TSH helped in revising it critically.

All authors read and approved the final manuscript.

## SUPPLEMENTARY MATERIALS FIGURES





## References

[1] Society AC (2016). Cancer Facts & Figures 2015.

[2] Ries LAG HD, Krapcho M, Mariotto A, Miller BA, Feuer EJ, Clegg L, Eisner MP, Horner MJ, Howlader N, Hayat M, Hankey BF, Edwards BK (2006). http://seer.cancer.gov/csr/1975_2003/, based on November 2005 SEER data submission. posted to the SEER web site. SEER Cancer Statistics Review, 1975-2003, National Cancer Institute.

[3] Callery MP, Chang KJ, Fishman EK, Talamonti MS, William Traverso L, Linehan DC (2009). Pretreatment assessment of resectable and borderline resectable pancreatic cancer: expert consensus statement. Annals of surgical oncology.

[4] Hazard L (2009). The role of radiation therapy in pancreas cancer. Gastrointestinal cancer research.

[5] Goodman KA, Hajj C (2013). Role of radiation therapy in the management of pancreatic cancer. J Surg Oncol.

[6] Roldan GE, Gunderson LL, Nagorney DM, Martin JK, Ilstrup DM, Holbrook MA, Kvols LK, McIlrath DC (1988). External beam versus intraoperative and external beam irradiation for locally advanced pancreatic cancer. Cancer.

[7] Oettle H, Post S, Neuhaus P, Gellert K, Langrehr J, Ridwelski K, Schramm H, Fahlke J, Zuelke C, Burkart C, Gutberlet K, Kettner E, Schmalenberg H, Weigang-Koehler K, Bechstein WO, Niedergethmann M (2007). Adjuvant chemotherapy with gemcitabine vs observation in patients undergoing curative-intent resection of pancreatic cancer: a randomized controlled trial. JAMA.

[8] Asiyanbola B, Gleisner A, Herman JM, Choti MA, Wolfgang CL, Swartz M, Edil BH, Schulick RD, Cameron JL, Pawlik TM (2009). Determining pattern of recurrence following pancreaticoduodenectomy and adjuvant 5-flurouracil-based chemoradiation therapy: effect of number of metastatic lymph nodes and lymph node ratio. J Gastrointest Surg.

[9] Kim J, Yu W, Kovalski K, Ossowski L (1998). Requirement for specific proteases in cancer cell intravasation as revealed by a novel semiquantitative PCR-based assay. Cell.

[10] Hanahan D, Weinberg RA (2000). The hallmarks of cancer. Cell.

[11] Hanahan D, Bergers G, Bergsland E (2000). Less is more, regularly: metronomic dosing of cytotoxic drugs can target tumor angiogenesis in mice. The Journal of clinical investigation.

[12] Varner JA, Cheresh DA (1996). Integrins and cancer. Current opinion in cell biology.

[13] Skubitz AP (2002). Adhesion molecules. Cancer treatment and research.

[14] Maurer CA, Friess H, Kretschmann B, Wildi S, Muller C, Graber H, Schilling M, Buchler MW (1998). Over-expression of ICAM-1, VCAM-1 and ELAM-1 might influence tumor progression in colorectal cancer. International journal of cancer Journal international du cancer.

[15] Fogar P, Basso D, Pasquali C, De Paoli M, Sperti C, Roveroni G, Pedrazzoli S, Plebani M (1997). Neural cell adhesion molecule (N-CAM) in gastrointestinal neoplasias. Anticancer research.

[16] Tempia-Caliera AA, Horvath LZ, Zimmermann A, Tihanyi TT, Korc M, Friess H, Buchler MW (2002). Adhesion molecules in human pancreatic cancer. Journal of surgical oncology.

[17] Matsuyama Y, Takao S, Aikou T (2002). Comparison of matrix metalloproteinase expression between primary tumors with or without liver metastasis in pancreatic and colorectal carcinomas. Journal of surgical oncology.

[18] Gress TM, Muller-Pillasch F, Lerch MM, Friess H, Buchler M, Adler G (1995). Expression and in-situ localization of genes coding for extracellular matrix proteins and extracellular matrix degrading proteases in pancreatic cancer. International journal of cancer Journal international du cancer.

[19] Billadeau DD, Chatterjee S, Bramati P, Sreekumar R, Shah V, Hedin K, Urrutia R (2006). Characterization of the CXCR4 signaling in pancreatic cancer cells. International journal of gastrointestinal cancer.

[20] Merati K, said Siadaty M, Andea A, Sarkar F, Ben-Josef E, Mohammad R, Philip P, Shields AF, Vaitkevicius V, Grignon DJ, Adsay NV (2001). Expression of inflammatory modulator COX-2 in pancreatic ductal adenocarcinoma and its relationship to pathologic and clinical parameters. American journal of clinical oncology.

[21] Tucker ON, Dannenberg AJ, Yang EK, Zhang F, Teng L, Daly JM, Soslow RA, Masferrer JL, Woerner BM, Koki AT, Fahey TJ (1999). Cyclooxygenase-2 expression is up-regulated in human pancreatic cancer. Cancer research.

[22] Okami J, Yamamoto H, Fujiwara Y, Tsujie M, Kondo M, Noura S, Oshima S, Nagano H, Dono K, Umeshita K, Ishikawa O, Sakon M, Matsuura N, Nakamori S, Monden M (1999). Overexpression of cyclooxygenase-2 in carcinoma of the pancreas. Clinical cancer research.

[23] Gurib-Fakim A (2006). Medicinal plants: traditions of yesterday and drugs of tomorrow. Molecular aspects of medicine.

[24] CG M., BM. R, Forest M, Balick J, Elisabetsky E, Laird S A (1996). Drug discovery and development at the National Cancer Institute: the role of natural products of plant origin. Medicinal Plant Resources of the Tropical.

[25] Mans DR, da Rocha AB, Schwartsmann G (2000). Anti-cancer drug discovery and development in Brazil: targeted plant collection as a rational strategy to acquire candidate anti-cancer compounds. The oncologist.

[26] Y Yoshie, W W, H P, T S (2002). Compositional difference of phenolic compounds between two seaweeds, Halimeda spp. Journal of Tokyo University Fisheries.

[27] E Furusawa, Furusawa S (1985). Anticancer activity of a natural product, viva-natural, extracted from Undaria pinnantifida on intraperitoneally implanted Lewis lung carcinoma. Oncology.

[28] Yuan YV, Carrington MF, Walsh NA (2005). Extracts from dulse (Palmaria palmata) are effective antioxidants and inhibitors of cell proliferation in vitro. Food and chemical toxicology.

[29] H Makita, T Tanaka, H Fujitsuka, N Tatematsu, K Satoh, A Hara, Mori H (1996). Chemoprevention of 4-nitroquinoline 1-oxide-induced rat oral carcinogenesis by the dietary flavonoids chalcone, 2-hydroxychalcone, and quercetin. Cancer research.

[30] Coombe DR, Parish CR, Ramshaw IA, Snowden JM (1987). Analysis of the inhibition of tumour metastasis by sulphated polysaccharides. International journal of cancer Journal international du cancer.

[31] Y Fujita, T Yamane, M Tanaka, K Kuwata, J Okuzumi, T Takahashi, H Fujiki, Okuda T (1989). Inhibitory effect of (-)-epigallocatechin gallate on carcinogenesis with N-ethyl-N’-nitro-N-nitrosoguanidine in mouse duodenum. Japanese journal of cancer research.

[32] T Tanaka, T Kojima, T Kawamori, A Wang, M Suzui, K Okamoto, Mori H (1993). Inhibition of 4-nitroquinoline-1-oxide-induced rat tongue carcinogenesis by the naturally occurring plant phenolics caffeic, ellagic, chlorogenic and ferulic acids. Carcinogenesis.

[33] Tanaka T (1994). Cancer chemoprevention by natural-products (review). Oncology reports.

[34] Zhang RL, Luo WD, Bi TN, Zhou SK (2012). Evaluation of antioxidant and immunity-enhancing activities of Sargassum pallidum aqueous extract in gastric cancer rats. Molecules.

[35] Yang JI, Yeh CC, Lee JC, Yi SC, Huang HW, Tseng CN, Chang HW (2012). Aqueous extracts of the edible Gracilaria tenuistipitata are protective against H(2)O(2)-induced DNA damage, growth inhibition, and cell cycle arrest. Molecules.

[36] F Liu, J Wang, Chang AK, B Liu, L Yang, Q Li, P Wang, Zou X (2012). Fucoidan extract derived from Undaria pinnatifida inhibits angiogenesis by human umbilical vein endothelial cells. Phytomedicine.

[37] Z Zhang, K Teruya, H Eto, Shirahata S (2011). Fucoidan extract induces apoptosis in MCF-7 cells via a mechanism involving the ROS-dependent JNK activation and mitochondria-mediated pathways. PloS one.

[38] Kim SK, Karagozlu MZ (2011). Marine algae: natural product source for gastrointestinal cancer treatment. Advances in food and nutrition research.

[39] Jayasooriya RG, Choi YH, Moon SK, Kim WJ, Kim GY (2012). Methanol extract of Hydroclathrus clathratus suppresses matrix metalloproteinase-9 in T24 bladder carcinoma cells by suppressing the NF-kappaB and MAPK pathways. Oncology reports.

[40] S Aravindan, Delma CR, Thirugnanasambandan SS, Herman TS, Aravindan N (2013). Anti-pancreatic cancer deliverables from sea: first-hand evidence on the efficacy, molecular targets and mode of action for multifarious polyphenols from five different brown-algae. PLoS One.

[41] S Aravindan, Ramraj SK, Somasundaram ST, Aravindan N (2015). Novel adjuvants from seaweed impede autophagy signaling in therapy-resistant residual pancreatic cancer. J Biomed Sci.

[42] S Aravindan, Ramraj SK, Somasundaram ST, Herman TS, Aravindan N (2015). Polyphenols from marine brown algae target radiotherapy-coordinated EMT and stemness-maintenance in residual pancreatic cancer. Stem cell research & therapy.

[43] Fidler IJ (2002). Critical determinants of metastasis. Semin Cancer Biol.

[44] Nguyen DX, Bos PD, Massague J (2009). Metastasis: from dissemination to organ-specific colonization. Nat Rev Cancer.

[45] A Stathis, Moore MJ (2010). Advanced pancreatic carcinoma: current treatment and future challenges. Nature reviews Clinical oncology.

[46] N Aravindan, Herman TS (2006). Mechanism of hyperthermia-induced radiosensitization of human breast cancer cells.

[47] N Aravindan, S Aravindan, Herman TS, Natarajan M (2013). EGFR tyrosine kinase inhibitor pelitinib regulates radiation-induced p65-dependent telomerase activation in squamous cell carcinoma. Radiat Res.

[48] N Aravindan, R Madhusoodhanan, S Ahmad, D Johnson, Herman TS (2008). Curcumin inhibits NFkappaB mediated radioprotection and modulate apoptosis related genes in human neuroblastoma cells. Cancer Biol Ther.

[49] N Aravindan, R Madhusoodhanan, M Natarajan, Herman TS (2008). Alteration of apoptotic signaling molecules as a function of time after radiation in human neuroblastoma cells. Mol Cell Biochem.

[50] N Aravindan, M Rakhesh, J Ambarish, Herman T (2007). Radio-adaptation mediated by persistent activation of NFκB through positive feed back (NFκB-TNFα-NFκB) cycle in neuroblastoma cells. AACR Annual Meeting.

[51] N Aravindan, Thomas CR, Aravindan S, Mohan AS, J Veeraraghavan, Natarajan M (2011). Irreversible EGFR Inhibitor EKB-569 targets low-LET gamma-radiation-triggered rel orchestration and potentiates cell death in squamous cell carcinoma. PloS one.

[52] N Aravindan, J Veeraraghavan, R Madhusoodhanan, Herman TS, Natarajan M (2011). Curcumin regulates low-linear energy transfer gamma-radiation-induced NFkappaB-dependent telomerase activity in human neuroblastoma cells. Int J Radiat Oncol Biol Phys.

[53] S Aravindan, M Natarajan, V Awasthi, Herman TS, Aravindan N (2013). Novel synthetic monoketone transmute radiation-triggered NFkappaB-dependent TNFalpha cross-signaling feedback maintained NFkappaB and favors neuroblastoma regression. PLoS One.

[54] S Aravindan, M Natarajan, Herman TS, Aravindan N (2013). Radiation-induced TNFalpha cross signaling-dependent nuclear import of NFkappaB favors metastasis in neuroblastoma. Clinical & experimental metastasis.

[55] S Aravindan, M Natarajan, Herman TS, V Awasthi, Aravindan N (2013). Molecular basis of ‘hypoxic’ breast cancer cell radio-sensitization: phytochemicals converge on radiation induced Rel signaling. Radiat Oncol.

[56] R Madhusoodhanan, M Natarajan, Singh JV, A Jamgade, V Awasthi, S Anant, Herman TS, Aravindan N (2010). Effect of black raspberry extract in inhibiting NFkappa B dependent radioprotection in human breast cancer cells. Nutrition and cancer.

[57] R Madhusoodhanan, M Natarajan, J Veeraraghavan, Herman TS, Aravindan N (2009). NFkappaB activity and transcriptional responses in human breast adenocarcinoma cells after single and fractionated irradiation. Cancer biology & therapy.

[58] J Veeraraghavan, M Natarajan, S Aravindan, Herman TS, Aravindan N (2011). Radiation-triggered tumor necrosis factor (TNF) alpha-NFkappaB cross-signaling favors survival advantage in human neuroblastoma cells. The Journal of biological chemistry.

[59] Zlotnik A (2004). Chemokines in neoplastic progression. Seminars in cancer biology.

[60] Balkwill F (2004). The significance of cancer cell expression of the chemokine receptor CXCR4. Seminars in cancer biology.

[61] M Kucia, K Jankowski, R Reca, M Wysoczynski, L Bandura, Allendorf DJ, J Zhang, J Ratajczak, Ratajczak MZ (2004). CXCR4-SDF-1 signalling, locomotion, chemotaxis and adhesion. Journal of molecular histology.

[62] A Muller, B Homey, H Soto, N Ge, D Catron, Buchanan ME, T McClanahan, E Murphy, W Yuan, Wagner SN, Barrera JL, A Mohar, E Verastegui, Zlotnik A (2001). Involvement of chemokine receptors in breast cancer metastasis. Nature.

[63] Z Wang, Q Ma, P Li, H Sha, X Li, Xu J (2013). Aberrant expression of CXCR4 and beta-catenin in pancreatic cancer. Anticancer research.

[64] Z Liang, Y Yoon, J Votaw, Goodman MM, L Williams, Shim H (2005). Silencing of CXCR4 blocks breast cancer metastasis. Cancer research.

[65] H Murata, S Kawano, S Tsuji, M Tsuji, H Sawaoka, Y Kimura, H Shiozaki, Hori M (1999). Cyclooxygenase-2 overexpression enhances lymphatic invasion and metastasis in human gastric carcinoma. The American journal of gastroenterology.

[66] S Gupta, M Srivastava, N Ahmad, Bostwick DG, Mukhtar H (2000). Over-expression of cyclooxygenase-2 in human prostate adenocarcinoma. The Prostate.

[67] H Wolff, K Saukkonen, S Anttila, A Karjalainen, H Vainio, Ristimaki A (1998). Expression of cyclooxygenase-2 in human lung carcinoma. Cancer research.

[68] Mohammed SI, Knapp DW, Bostwick DG, Foster RS, Khan KN, Masferrer JL, Woerner BM, Snyder PW, Koki AT (1999). Expression of cyclooxygenase-2 (COX-2) in human invasive transitional cell carcinoma (TCC) of the urinary bladder. Cancer research.

[69] D Hwang, D Scollard, J Byrne, Levine E (1998). Expression of cyclooxygenase-1 and cyclooxygenase-2 in human breast cancer. Journal of the National Cancer Institute.

[70] Smith WL, DeWitt DL, Garavito RM (2000). Cyclooxygenases: structural, cellular, and molecular biology. Annual review of biochemistry.

[71] S Pugh, Thomas GA (1994). Patients with adenomatous polyps and carcinomas have increased colonic mucosal prostaglandin E2. Gut.

[72] Rolland PH, Martin PM, J Jacquemier, Rolland AM, Toga M (1980). Prostaglandin in human breast cancer: Evidence suggesting that an elevated prostaglandin production is a marker of high metastatic potential for neoplastic cells. Journal of the National Cancer Institute.

[73] K Uefuji, T Ichikura, Mochizuki H (2000). Cyclooxygenase-2 expression is related to prostaglandin biosynthesis and angiogenesis in human gastric cancer. Clinical cancer research.

[74] H Sheng, J Shao, Washington MK, DuBois RN (2001). Prostaglandin E2 increases growth and motility of colorectal carcinoma cells. The Journal of biological chemistry.

[75] M Tsujii, S Kawano, DuBois RN Cyclooxygenase-2 expression in human colon cancer cells increases metastatic potential.

[76] M Tsujii, DuBois RN (1995). Alterations in cellular adhesion and apoptosis in epithelial cells overexpressing prostaglandin endoperoxide synthase 2. Cell.

[77] M Tsujii, S Kawano, S Tsuji, H Sawaoka, M Hori, DuBois RN (1998). Cyclooxygenase regulates angiogenesis induced by colon cancer cells. Cell.

[78] Clevers H (2006). Wnt/beta-catenin signaling in development and disease. Cell.

[79] F Yang, Q Zeng, G Yu, S Li, Wang CY (2006). Wnt/beta-catenin signaling inhibits death receptor-mediated apoptosis and promotes invasive growth of HNSCC. Cellular signalling.

[80] White BD, Chien AJ, Dawson DW (2012). Dysregulation of Wnt/beta-catenin signaling in gastrointestinal cancers. Gastroenterology.

[81] A Saadeddin, Babaei-Jadidi R, Spencer-Dene B, Nateri AS (2009). The links between transcription, beta-catenin/JNK signaling, and carcinogenesis. Molecular cancer research.

[82] J Pujal, G Capella, Real FX (2006). The Wnt pathway is active in a small subset of pancreas cancer cell lines. Biochimica et biophysica acta.

[83] X Yu, Y Wang, DeGraff DJ, Wills ML, Matusik RJ (2011). Wnt/beta-catenin activation promotes prostate tumor progression in a mouse model. Oncogene.

[84] Hsu HP, Shan YS, Jin YT, Lai MD, Lin PW (2010). Loss of E-cadherin and beta-catenin is correlated with poor prognosis of ampullary neoplasms. Journal of surgical oncology.

[85] Lee HS, Park MH, Yang SJ, Park KC, Kim NS, Kim YS, Kim DI, Yoo HS, Choi EJ, Yeom YI (2007). Novel candidate targets of Wnt/beta-catenin signaling in hepatoma cells. Life sciences.

[86] L Wang, H Cheng, Y Liu, W Yu, G Zhang, B Chen, Z Yu, Hu S (2011). Prognostic value of nuclear beta-catenin overexpression at invasive front in colorectal cancer for synchronous liver metastasis. Annals of surgical oncology.

[87] R Nishimura, T Osako, Y Nishiyama, R Tashima, M Nakano, M Fujisue, Y Toyozumi, Arima N (2014). Prognostic significance of Ki-67 index value at the primary breast tumor in recurrent breast cancer. Molecular and clinical oncology.

[88] P Gong, Y Wang, G Liu, J Zhang, Wang Z (2013). New insight into Ki67 expression at the invasive front in breast cancer. PLoS One.

[89] S Kebache, J Ash, Annis MG, J Hagan, M Huber, J Hassard, Stewart CL, M Whiteway, Nantel A (2007). Grb10 and active Raf-1 kinase promote Bad-dependent cell survival. The Journal of biological chemistry.

[90] F Liu, Roth RA Grb-IR: a SH2-domain-containing protein that binds to the insulin receptor and inhibits its function.

[91] O’Neill TJ, Rose DW, Pillay TS, K Hotta, Olefsky JM, Gustafson TA (1996). Interaction of a GRB-IR splice variant (a human GRB10 homolog) with the insulin and insulin-like growth factor I receptors. Evidence for a role in mitogenic signaling. The Journal of biological chemistry.

[92] Stein EG, Gustafson TA, Hubbard SR (2001). The BPS domain of Grb10 inhibits the catalytic activity of the insulin and IGF1 receptors. FEBS letters.

[93] Wick KR, Werner ED, P Langlais, Ramos FJ, Dong LQ, Shoelson SE, Liu F (2003). Grb10 inhibits insulin-stimulated insulin receptor substrate (IRS)-phosphatidylinositol 3-kinase/Akt signaling pathway by disrupting the association of IRS-1/IRS-2 with the insulin receptor. The Journal of biological chemistry.

[94] Morrione A (2003). Grb10 adapter protein as regulator of insulin-like growth factor receptor signaling. Journal of cellular physiology.

[95] A Morrione, B Valentinis, M Resnicoff, S Xu, Baserga R (1997). The role of mGrb10alpha in insulin-like growth factor I-mediated growth. The Journal of biological chemistry.

[96] P Langlais, Dong LQ, Ramos FJ, D Hu, Y Li, Quon MJ, Liu F (2004). Negative regulation of insulin-stimulated mitogen-activated protein kinase signaling by Grb10. Mol Endocrinol.

[97] J Veeraraghavan, M Natarajan, P Lagisetty, V Awasthi, Herman TS, Aravindan N (2011). Impact of curcumin, raspberry extract, and neem leaf extract on rel protein-regulated cell death/radiosensitization in pancreatic cancer cells. Pancreas.

[98] N Aravindan, M Natarajan, Shaw AD (2006). Fenoldopam inhibits nuclear translocation of nuclear factor kappa B in a rat model of surgical ischemic acute renal failure. Journal of cardiothoracic and vascular anesthesia.

[99] N Aravindan, S Aravindan, Riedel BJ, Weng HR, Shaw AD (2007). Furosemide prevents apoptosis and associated gene expression in a rat model of surgical ischemic acute renal failure. Renal failure.

[100] N Aravindan, S Aravindan, K Shanmugasundaram, Shaw AD (2007). Periods of systemic partial hypoxia induces apoptosis and inflammation in rat skeletal muscle. Molecular and cellular biochemistry.

[101] N Aravindan, Cata JP, Dougherty PM, Shaw AD (2006). Effect of fenoldopam on ischemia/reperfusion-induced apoptosis. Renal failure.

[102] N Aravindan, Cata JP, L Hoffman, Dougherty PM, Riedel BJ, Price KJ, Shaw AD (2006). Effects of isoflurane, pentobarbital, and urethane on apoptosis and apoptotic signal transduction in rat kidney. Acta anaesthesiologica Scandinavica.

[103] S Aravindan, M Natarajan, Ramraj SK, V Pandian, Khan FH, Herman TS, Aravindan N (2014). Abscopal effect of low-LET gamma-radiation mediated through Rel protein signal transduction in a mouse model of nontargeted radiation response. Cancer gene therapy.

[104] J Veeraraghavan, M Natarajan, Herman TS, Aravindan N (2011). Low-dose gamma-radiation-induced oxidative stress response in mouse brain and gut: regulation by NFkappaB-MnSOD cross-signaling. Mutat Res.

[105] Khan FH, V Pandian, Ramraj SK, S Aravindan, M Natarajan, S Azadi, Herman TS, Aravindan N (2015). RD3 loss dictates high-risk aggressive neuroblastoma and poor clinical outcomes. Oncotarget.

