# Supplementary material for: Hormophysa triquerta polyphenol, an elixir that deters CXCR4- and COX2-dependent dissemination destiny of treatment-resistant pancreatic cancer cells
Source: Oncotarget. 2016 Dec 10;8(4):5717–34. doi: 10.18632/oncotarget.13900 (PMC5351584; doi:10.18632/oncotarget.13900)
Supplement: Supplementary file 1 [file oncotarget-08-5717-s001.pdf]

# *Hormophysa triquerta* polyphenol, an elixir that deters CXCR4- and COX2-dependent dissemination destiny of treatment-resistant pancreatic cancer cells

**Supplementary Material**

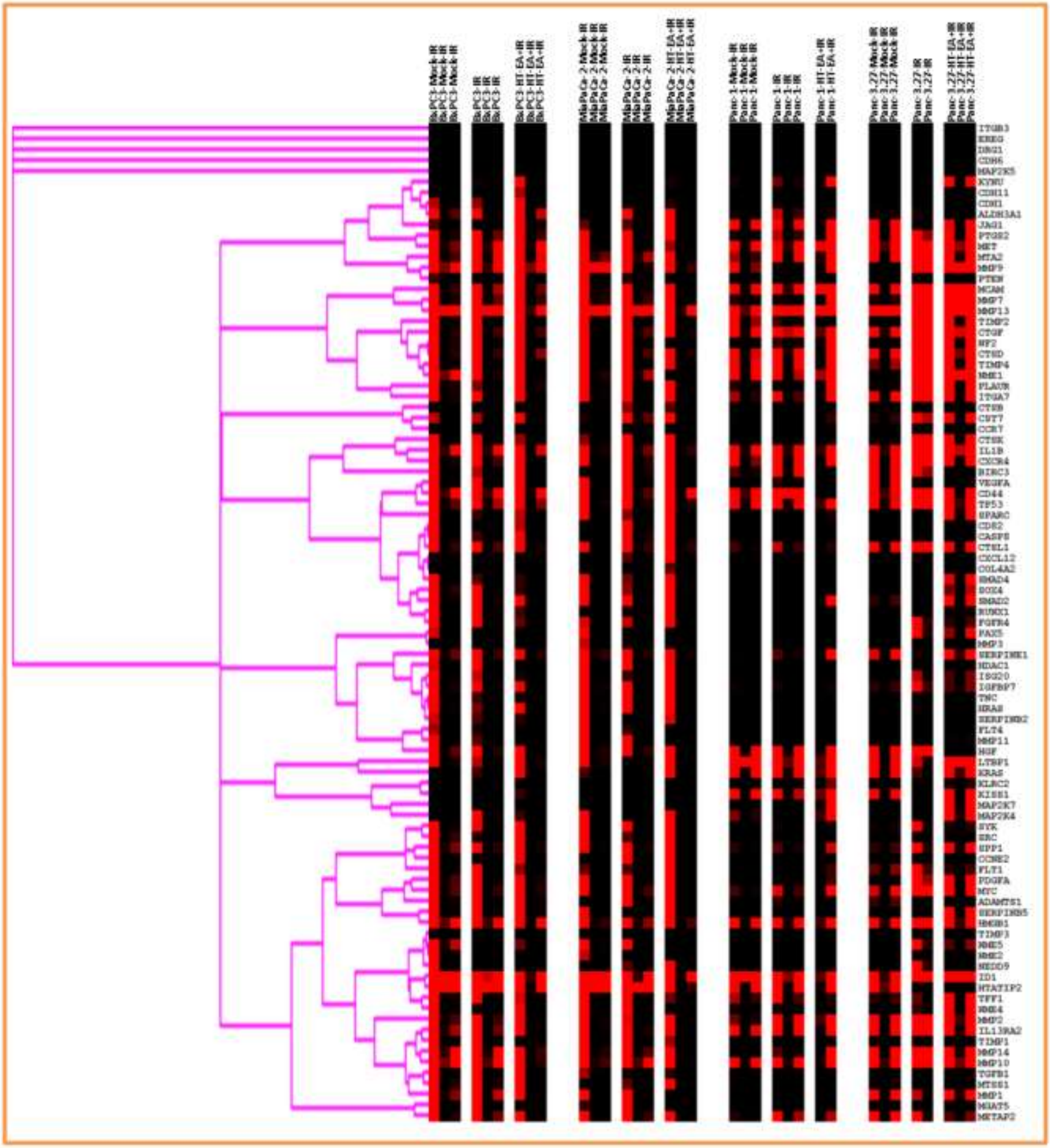


**Figure S1:** Heatmap color representation of relative expression levels of 93 tumor invasion and metastasis signaling molecules in human pancreatic cancer (BXPC-3, MiaPaCa-2, Panc-1, Panc-3.27) cells either mock-irradiated or exposed to radiation, with or without HT-EA pre-treatment.
