# Supplementary material for: Hormophysa triquerta polyphenol, an elixir that deters CXCR4- and COX2-dependent dissemination destiny of treatment-resistant pancreatic cancer cells
Source: Oncotarget. 2016 Dec 10;8(4):5717–34. doi: 10.18632/oncotarget.13900 (PMC5351584; doi:10.18632/oncotarget.13900)
Supplement: Supplementary file 2 [file oncotarget-08-5717-s002.docx]

**Table S1.** List of tumor invasion and metastasis (TIM) related transcripts archived in the QPCR profiling, and their location and types

| **Symbol** | **Entrez Gene Name** | **Location** | **Type(s)** |
| --- | --- | --- | --- |
| ADAMTS1 | ADAM metallopeptidase with thrombospondin type 1 motif, 1 | Extracellular Space | peptidase |
| ALDH3A1 | aldehyde dehydrogenase 3 family, member A1 | Cytoplasm | enzyme |
| BIRC3 | baculoviral IAP repeat containing 3 | Cytoplasm | enzyme |
| CASP8 | caspase 8, apoptosis-related cysteine peptidase | Nucleus | peptidase |
| CCNE2 | cyclin E2 | Nucleus | other |
| CCR7 | chemokine (C-C motif) receptor 7 | Plasma Membrane | G-protein coupled receptor |
| CD44 | CD44 molecule (Indian blood group) | Plasma Membrane | enzyme |
| CD82 | CD82 molecule | Plasma Membrane | other |
| CDH1 | cadherin 1, type 1, E-cadherin (epithelial) | Plasma Membrane | other |
| CDH11 | cadherin 11, type 2, OB-cadherin (osteoblast) | Plasma Membrane | other |
| CDH6 | cadherin 6, type 2, K-cadherin (fetal kidney) | Plasma Membrane | other |
| COL4A2 | collagen, type IV, alpha 2 | Extracellular Space | other |
| CST7 | cystatin F (leukocystatin) | Extracellular Space | other |
| CTGF | connective tissue growth factor | Extracellular Space | growth factor |
| CTSB | cathepsin B | Cytoplasm | peptidase |
| CTSD | cathepsin D | Cytoplasm | peptidase |
| CTSK | cathepsin K | Cytoplasm | peptidase |
| CTSL | cathepsin L | Cytoplasm | peptidase |
| CXCL12 | chemokine (C-X-C motif) ligand 12 | Extracellular Space | cytokine |
| CXCR4 | chemokine (C-X-C motif) receptor 4 | Plasma Membrane | G-protein coupled receptor |
| DRG1 | developmentally regulated GTP binding protein 1 | Cytoplasm | other |
| EREG | epiregulin | Extracellular Space | growth factor |
| FGFR4 | fibroblast growth factor receptor 4 | Plasma Membrane | kinase |
| FLT1 | fms-related tyrosine kinase 1 | Plasma Membrane | kinase |
| FLT4 | fms-related tyrosine kinase 4 | Plasma Membrane | transmembrane receptor |
| HDAC1 | histone deacetylase 1 | Nucleus | transcription regulator |
| HGF | hepatocyte growth factor (hepapoietin A; scatter factor) | Extracellular Space | growth factor |
| HMGB1 | high mobility group box 1 | Nucleus | transcription regulator |
| HRAS | Harvey rat sarcoma viral oncogene homolog | Plasma Membrane | enzyme |
| HTATIP2 | HIV-1 Tat interactive protein 2, 30kDa | Nucleus | transcription regulator |
| ID1 | inhibitor of DNA binding 1, dominant negative helix-loop-helix protein | Nucleus | transcription regulator |
| IGFBP7 | insulin-like growth factor binding protein 7 | Extracellular Space | transporter |
| IL13RA2 | interleukin 13 receptor, alpha 2 | Plasma Membrane | transmembrane receptor |
| IL1B | interleukin 1, beta | Extracellular Space | cytokine |
| ISG20 | interferon stimulated exonuclease gene 20kDa | Nucleus | enzyme |
| ITGA7 | integrin, alpha 7 | Plasma Membrane | other |
| ITGB3 | integrin, beta 3 (platelet glycoprotein IIIa, antigen CD61) | Plasma Membrane | transmembrane receptor |
| JAG1 | jagged 1 | Extracellular Space | growth factor |
| KISS1 | KiSS-1 metastasis-suppressor | Cytoplasm | other |
| KLRC2 | killer cell lectin-like receptor subfamily C, member 2 | Plasma Membrane | transmembrane receptor |
| KRAS | Kirsten rat sarcoma viral oncogene homolog | Cytoplasm | enzyme |
| KYNU | kynureninase | Cytoplasm | enzyme |
| LTBP1 | latent transforming growth factor beta binding protein 1 | Extracellular Space | other |
| MAP2K4 | mitogen-activated protein kinase kinase 4 | Cytoplasm | kinase |
| MAP2K5 | mitogen-activated protein kinase kinase 5 | Cytoplasm | kinase |
| MAP2K7 | mitogen-activated protein kinase kinase 7 | Cytoplasm | kinase |
| MCAM | melanoma cell adhesion molecule | Plasma Membrane | other |

| **Symbol** | **Entrez Gene Name** | **Location** | **Type(s)** |
| --- | --- | --- | --- |
| MET | met proto-oncogene | Plasma Membrane | kinase |
| METAP2 | methionyl aminopeptidase 2 | Cytoplasm | peptidase |
| MGAT5 | mannosyl (alpha-1,6-)-glycoprotein beta-1,6-N-acetyl-glucosaminyltransferase | Cytoplasm | enzyme |
| MMP1 | matrix metallopeptidase 1 (interstitial collagenase) | Extracellular Space | peptidase |
| MMP10 | matrix metallopeptidase 10 (stromelysin 2) | Extracellular Space | peptidase |
| MMP11 | matrix metallopeptidase 11 (stromelysin 3) | Extracellular Space | peptidase |
| MMP13 | matrix metallopeptidase 13 (collagenase 3) | Extracellular Space | peptidase |
| MMP14 | matrix metallopeptidase 14 (membrane-inserted) | Extracellular Space | peptidase |
| MMP2 | matrix metallopeptidase 2 (gelatinase A, 72kDa gelatinase, 72kDa type IV collagenase) | Extracellular Space | peptidase |
| MMP3 | matrix metallopeptidase 3 (stromelysin 1, progelatinase) | Extracellular Space | peptidase |
| MMP7 | matrix metallopeptidase 7 (matrilysin, uterine) | Extracellular Space | peptidase |
| MMP9 | matrix metallopeptidase 9 (gelatinase B,, 92kDa type IV collagenase) | Extracellular Space | peptidase |
| MTA2 | metastasis associated 1 family, member 2 | Nucleus | transcription regulator |
| MTSS1 | metastasis suppressor 1 | Cytoplasm | other |
| MYC | v-myc avian myelocytomatosis viral oncogene homolog | Nucleus | transcription regulator |
| NEDD9 | neural precursor cell expressed, developmentally down-regulated 9 | Nucleus | other |
| NF2 | neurofibromin 2 (merlin) | Plasma Membrane | other |
| NME1 | NME/NM23 nucleoside diphosphate kinase 1 | Cytoplasm | kinase |
| NME2 | NME/NM23 nucleoside diphosphate kinase 2 | Nucleus | kinase |
| NME4 | NME/NM23 nucleoside diphosphate kinase 4 | Cytoplasm | kinase |
| NME5 | NME/NM23 family member 5 | Other | kinase |
| PAX5 | paired box 5 | Nucleus | transcription regulator |
| PDGFA | platelet-derived growth factor alpha polypeptide | Extracellular Space | growth factor |
| PLAUR | plasminogen activator, urokinase receptor | Plasma Membrane | transmembrane receptor |
| PTEN | phosphatase and tensin homolog | Cytoplasm | phosphatase |
| PTGS2 | prostaglandin-endoperoxide synthase 2 (prostaglandin G/H synthase and cyclooxygenase) | Cytoplasm | enzyme |
| RUNX1 | runt-related transcription factor 1 | Nucleus | transcription regulator |
| SERPINB2 | serpin peptidase inhibitor, clade B (ovalbumin), member 2 | Extracellular Space | other |
| SERPINB5 | serpin peptidase inhibitor, clade B (ovalbumin), member 5 | Extracellular Space | other |
| SERPINE1 | serpin peptidase inhibitor, clade E (nexin, plasminogen activator inhibitor type 1), member 1 | Extracellular Space | other |
| SMAD2 | SMAD family member 2 | Nucleus | transcription regulator |
| SMAD4 | SMAD family member 4 | Nucleus | transcription regulator |
| SOX4 | SRY (sex determining region Y)-box 4 | Nucleus | transcription regulator |
| SPARC | secreted protein, acidic, cysteine-rich (osteonectin) | Extracellular Space | other |
| SPP1 | secreted phosphoprotein 1 | Extracellular Space | cytokine |
| SRC | v-src avian sarcoma (Schmidt-Ruppin A-2) viral oncogene homolog | Cytoplasm | kinase |
| SYK | spleen tyrosine kinase | Cytoplasm | kinase |
| TFF1 | trefoil factor 1 | Extracellular Space | other |
| TGFB1 | transforming growth factor, beta 1 | Extracellular Space | growth factor |
| TIMP1 | TIMP metallopeptidase inhibitor 1 | Extracellular Space | other |
| TIMP2 | TIMP metallopeptidase inhibitor 2 | Extracellular Space | other |
| TIMP3 | TIMP metallopeptidase inhibitor 3 | Extracellular Space | other |
| TIMP4 | TIMP metallopeptidase inhibitor 4 | Extracellular Space | other |
| TNC | tenascin C | Extracellular Space | other |
| TP53 | tumor protein p53 | Nucleus | transcription regulator |
| VEGFA | vascular endothelial growth factor A | Extracellular Space | growth factor |
